# QT Dispersion and Drug-Induced Torsade de Pointes

**DOI:** 10.7759/cureus.12895

**Published:** 2021-01-25

**Authors:** Ari Friedman, Jeremy Miles, Jared Liebelt, Panagiota Christia, Krysthel Engstrom, Rosy Thachil, Michael Grushko, Robert T Faillace

**Affiliations:** 1 Medicine, Jacobi Medical Center/Albert Einstein College of Medicine, Bronx, USA; 2 Cardiology, Montefiore Medical Center, Bronx, USA; 3 Cardiology, North Shore University Health Systems-Metro Chicago, Chicago, USA; 4 Cardiology, Mt. Sinai Medical Center, New York, USA; 5 Cardiology, Albert Einstein College of Medicine/Jacobi Medical Center, Bronx, USA; 6 Medicine, Albert Einstein College of Medicine/Jacobi Medical Center, Bronx, USA

**Keywords:** arrhythmia, torsade de pointes, qt dispersion, early after depolarizations, antiarrhythmic agents

## Abstract

Background

Amiodarone causes less drug-induced torsade de pointes (TdP) compared to other class III antiarrhythmics. Two theories proposed for this finding include that amiodarone has less repolarization heterogeneity, and/or decreases early after depolarization (EADs). Corrected QT (QTc) dispersion as measured on a surface electrocardiogram (ECG) represents spatial heterogeneity of ventricular repolarization.

Objective

The purpose of this study was to analyze the difference in QT dispersion between amiodarone and other class III antiarrhythmics and to determine the etiology of TdP.

Methods

This was a retrospective, observational study at Montefiore Medical Center between January 2005 and January 2015. Inclusion criteria were adults >18 years on amiodarone, dofetilide, or sotalol with prolonged QT interval on 12-lead ECG. ECGs were reviewed by three blinded observers. QTc was calculated using the Bazett and Framingham formulas. QTc dispersion was calculated by subtracting the shortest from the longest QTc. Analysis of variance (ANOVA) was applied for comparison between antiarrhythmic groups with Bonferroni correction for multiple comparisons.

Results

A total of 447 ECGs were reviewed and 77 ECGs met inclusion criteria. The average QT dispersion for amiodarone, dofetilide, and sotalol was 0.050, 0.037, and 0.034, respectively (p=0.006) and the average QTc dispersion by Bazett was 0.053, 0.038, and 0.037 (p=0.008) and by Framingham was 0.049, 0.036, and 0.035 (p=0.009), respectively.

Conclusion

Our results show that given the increase in QT dispersion seen with amiodarone, heterogeneous ventricular repolarization as measured by QTc dispersion likely does not account for the lower incidence of drug-induced TdP seen with amiodarone. The ability of amiodarone to decrease EADs via sodium-channel blockade is more likely the explanation for its lower incidence of drug-induced TdP.

## Introduction

QT dispersion, defined as the difference between the largest and smallest QT intervals in all the leads of a surface electrocardiogram (ECG), has been studied extensively over the past 30 years [1]. It is theorized that QT dispersion on an ECG signifies regional differences in ventricular repolarization and, therefore, increased QT dispersion reflects greater heterogeneity of myocyte repolarization, portending a greater risk of ventricular arrhythmia [1]. Despite the multitude of studies, the significance and clinical impact of QT dispersion measurement in different study populations remain controversial, with studies illustrating conflicting results [2-4].

Certain medications, such as the Vaughn-Williams Class III anti-arrhythmic agents, including sotalol, dofetilide, and amiodarone, are known to cause a prolonged QT interval and a consequent increased risk of ventricular arrhythmia [5]. However, the relationship between the QT-prolonging effect and QT dispersion of these agents has not been fully elucidated, and studies have shown conflicting evidence regarding their effects. Some previous studies have demonstrated that while sotalol and amiodarone may cause an increased QT interval, they may actually reduce QT dispersion [6-7]. Other studies have concluded that only amiodarone reduces QT dispersion while sotalol and dofetilide do not [8-10]. The range of studies also includes those that have shown that amiodarone does not reduce QT dispersion [11-12]. This study expands on the current knowledge base by investigating the effects of Class III antiarrhythmics, sotalol, dofetilide, and amiodarone, on ECG QT dispersion and in a multi-ethnic cohort.

The debate over amiodarone’s effect on QT dispersion is significant due to the paradoxical finding that while amiodarone is observed to increase the QT interval, which is associated with pro-arrhythmic effects and fatal ventricular arrhythmias like Torsade de Pointes (TdP), it still has an observed low incidence of TdP [13]. Analysis and comparison of the effect of different class III antiarrhythmic agents on QT dispersion can help further understand the mechanism of this paradoxical effect of amiodarone: whether the low incidence of TdP by amiodarone despite its QT-prolonging effect is mediated by increasing homogeneous ventricular repolarization or by its effects on reducing early after-depolarizations (EADs) [14-16].

## Materials and methods

Patient selection

A retrospective chart review was performed on consecutive patients who presented for inpatient or outpatient care at Montefiore Medical Center from January 2005 through January 2015 with the following inclusion criteria: age greater than 18 at the time of presentation, being prescribed either sotalol, dofetilide, or amiodarone, and having evidence of a prolonged QT interval on a 12-lead surface ECG at the time of medication use. A prolonged QT interval was defined as 450 milliseconds (ms) or higher in males and 470 ms or higher in females. Patients were required to be taking the respective medication for at least 24 hours prior to the ECG obtained for analysis. Exclusion criteria included those with congenital long QT syndrome, those with technically poor baseline ECGs preventing accurate interval measurements, ventricular paced rhythms, pre-excitation with a QRS duration >110 milliseconds, a right bundle or left bundle branch block, bradyarrhythmias with heart rates of less than 50, or tachyarrhythmias greater than 100 beats per minute.

Study data were collected using the hospital’s electronic medical record query database, Clinical Looking Glass^TM ^(Clinical Looking Glass (CLG), Emerging Health Information Technology, Yonkers, New York). Collected data included baseline demographics, comorbidities, electrolyte, and creatinine values at the time of study ECG, and the most recent ejection fraction (EF) on transthoracic echocardiogram (TTE). Patients were divided into three groups based on the Class III anti-arrhythmic prescribed: sotalol, dofetilide, and amiodarone.

ECG analysis

MUSE® Cardiology Information System (GE Healthcare, Chicago, Illinois) was used to identify qualifying ECGs. Technically high-quality 12-lead ECGs were required for this study. ECGs were standardized at the normal speed of 25 millimeters/second with an amplitude of 10mm/mV. MUSE® utilizes the Marquette 12SL ECG analysis software (GE Healthcare) for 12 lead ECG analysis and derivation of QTc intervals. Each ECG was evaluated by three blinded investigators: two internal medicine residents and one board-certified cardiologist and was analyzed for the baseline QT interval, the corrected QT (QTc) interval, the measured QT dispersion, and the corrected QT (QTc) dispersion. The QT interval was measured from the earliest QRS deflection to the end of the T wave, which was defined by the return of the T wave to the isoelectric baseline. If U waves were present, the end of the T wave was estimated to the point of return to isoelectric baseline at the judgment of the ECG interpreter. Measurements obtained included the shortest and longest QT intervals on a 12-lead ECG as well as the preceding R-R interval of the corresponding measurement. The QTc interval was calculated using Bazett’s formula (QTc = QT/√RR) as well as the Framingham formula (QTc = QT + 1.75 (heart rate - 60)). QT dispersion was manually calculated by determining the difference in milliseconds between the maximum QT interval and the minimum QT interval on the 12-lead ECG during ectopic-free sinus rhythm.

Statistical analysis

Baseline characteristics were summarized using descriptive statistics, including mean (SD) or median (IQR) for continuous variables and percentage for categorical variables. Baseline continuous variables were compared between groups using the t-test or nonparametric equivalent, and the chi-square test was employed for a comparison of categorical variables. The analysis of variance (ANOVA) was used for comparison between groups with Bonferroni correction for multi-comparison analysis. Average QT and QTc measurements for each of the three observers were calculated for each individual antiarrhythmic medication. The average QT and QTc dispersion for all observers was calculated according to each antiarrhythmic medication group in order to decrease the variance. The correlation coefficient (r) was calculated to compare individual measurements, and the interclass correlation coefficient (ICC) was calculated using a two-way random-effects model. Statistical significance was determined at an alpha of ≤ 0.05. All analyses were performed in the Statistical Package for the Social Sciences (SPSS) v21 (IBM Corp., Armonk, NY).

## Results

Baseline data

A total of 447 patients were initially identified, and after applying exclusion criteria, 77 patients were included in the final analysis. 26 patients were prescribed amiodarone, 26 dofetilide, and 25 sotalol. As shown in Table 1, the average age of the included patients was 63.1 +/- 13.0, 54% were male, and each group was ethnically diverse. Those taking amiodarone compared to those on either sotalol or dofetilide had a higher rate of prior myocardial infarctions (MI) (38.5% vs 15.4% vs 12.5%, p<0.05), heart failure with a reduced ejection fraction (HFrEF) (42.3% vs 38.5% vs 12%, p=0.04), and chronic kidney disease (CKD) (57.7% vs 23.1% vs 20%, p<0.01). There was no difference in the rates of individuals with coronary artery disease, right ventricular dysplasia, heart failure with preserved ejection fraction (HFpEF), prior stroke, human immunodeficiency virus (HIV), or connective tissue diseases (CTD) among the groups (Table 1). Notably, there was no difference in electrolyte levels and left ventricular ejection fraction among the groups. However, those on amiodarone had higher baseline creatinine levels as compared to those on sotalol and dofetilide (3.4+3.0 vs 1+0.3 vs 1.2 +0.8).

**Table 1:** Demographics † Statistically significant Coronary artery disease, CAD; Myocardial infarction, MI; Heart failure with preserved ejection fraction, HFpEF; Heart failure with reduced ejection fraction, HFrEF; Cerebral vascular accident, CVA; Human immunodeficiency virus, HIV; Adult immunodeficiency disease, AIDS; Connective tissue disease, CTD; Chronic kidney disease, CKD; Left ventricular ejection fraction, LVEF

Total (N=77)	Amiodarone (N=26)	Dofetilide (N=26)	Sotalol (N=25)	
	(N or +/- SD)	(N or +/- SD)	(N or +/- SD)	p value
Age (years)	65.4 +/- 13.2	60.7 +/- 12.4	63.2 +/- 13.9	0.45
Male Gender (%)	57.7 (15)	53.8 (14)	48 (12)	0.78
Ethnicity (%)				0.49
-White	19.2 (5)	19.2 (5)	52 (13)	
-Hispanic	26.9 (7)	26.9 (7)	4 (1)	
-Black	30.8 (8)	19.2 (5)	16 (4)	
-Other	23.1 (6)	34.6(9)	28 (7)	
Comorbidities (%)				
-RV Dysplasia	0 (0)	0 (0)	1 (4)	0.59
-CAD	61.5 (16)	34.6 (9)	32 (8)	0.06
-MI	38.5 (10)	15.4 (4)	12.5 (3)	0.05†
-HFpEF	11.5 (3)	19.2 (5)	4 (1)	0.24
-HFrEF	42.3 (11)	38.5 (10)	12 (3)	0.04†
-CVA	19.2 (5)	15.4 (4)	4 (1)	0.24
-HIV/AIDs	7.7 (2)	0 (0)	0 (0)	0.13
-CTD	3.8 (1)	15.4 (4)	4 (1)	0.2
-CKD	57.7 (15)	23.1 (6)	20 (5)	< 0.01†
Laboratory Values				
-LVEF (%)	51 +/- 15	53 +/- 16	58 +/- 8	0.23
-Creatinine (mg/dL)	3.4 +/- 3.0	1 +/- 0.3	1.2 +/- 0.8	< 0.01†
-Sodium (mEq/L)	139.2 +/- 2.8	138 +/- 2.9	138.4 +/- 4.1	0.45
-Potassium (mEq/L)	4.2 +/- 0.5	4.1 +/- 0.4	4.3 +/- 0.4	0.37
-Calcium (mg/dL)	8.6 +/- 0.9	9 +/- 0.5	8.9 +/- 0.7	0.09
-Magnesium (mEq/L)	2 +/- 0.2	2 +/- 0.2	2 +/- 0.3	0.78

QT interval and QTc interval

ECG analysis of all 77 patients was performed by three blinded observers. There was no statistical difference in the mean QT and QTc measurements between antiarrhythmic groups when comparing measurements between observers. When the manual QT and QTc interval measurements for each anti-arrhythmic group were averaged among all three observers, there was a significant correlation to the computer-derived measurement. As shown in Table 2, the correlation coefficient (r) for the average of all observers’ manual QT interval compared to computer-generated measurement was 0.87 for amiodarone, 0.97 for dofetilide, and 0.92 for sotalol. There was a similar correlation between the averages of manual measurements of all observers for each antiarrhythmic when using both the Bazett and Framingham formulas as compared to the computer-derived QTc, with an (r) value for the Bazett formula of 0.72 for amiodarone, 0.84 for dofetilide, and 0.93 for sotalol. With the Framingham formula, there was an (r) value of 0.74 in the amiodarone group, 0.72 for dofetilide, and 0.83 for sotalol (Table 2).

**Table 2:** Correlation between manual and computer-generated QT measurements † Statistically significant (p < 0.01)

QT Interval	
Medication	Correlation Coefficient (r)
-Amiodarone	0.87
-Dofetilide	0.97
-Sotalol	0.97
-Overall	0.92
QTc Interval - Bazett	
Medication	Correlation Coefficient (r)
-Amiodarone	0.72
-Dofetilide	0.84
-Sotalol	0.93
-Overall	0.79
QTc Interval - Framingham	
Medication	Correlation Coefficient (r)
-Amiodarone	0.74
-Dofetilide	0.72
-Sotalol	0.83
-Overall	0.79

QT and QTc dispersion

**Figure 1:**
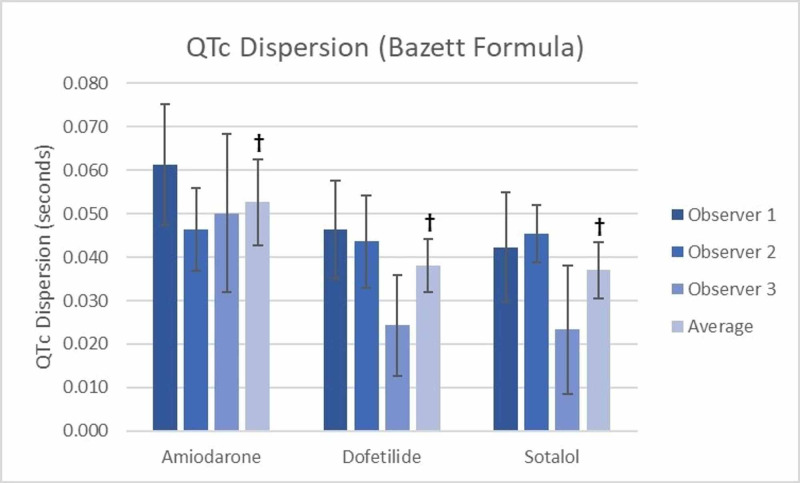
QTc Dispersion (Bazett Formula) Between Groups † Denotes a significant difference between groups with p=0.008 ‡ Denotes a significant difference in QTc dispersion between observer 2 and observer 3

**Figure 2:**
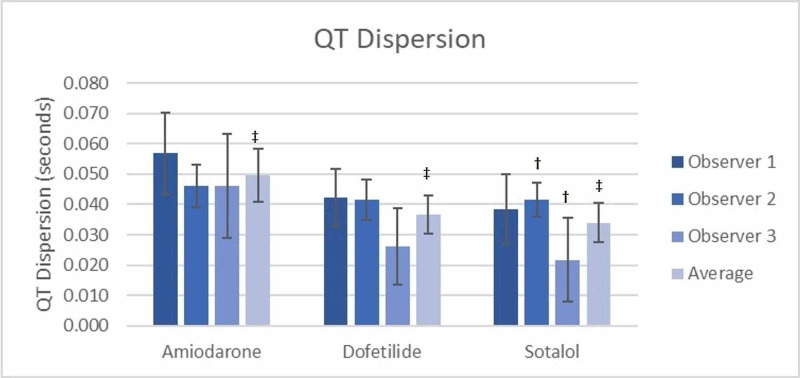
QTc Dispersion (Framingham Formula) Between Groups † Denotes a significant difference between groups with p=0.009 ‡ Denotes a significant difference in QTc dispersion between observer 2 and observer 3

All QT and QTc dispersion measurements are shown in Table 3. The inter-observer differences for both QT and QTc dispersion measurements were calculated and showed that there was no significant difference between observers measuring QT or QTc dispersion for both the amiodarone and dofetilide groups (Table 3). However, in the sotalol group, there was a significant difference between observers 2 and 3 regarding both QT (0.042 sec vs 0.022 sec) and QTc dispersion measurements (0.045 sec vs 0.023 sec using the Bazett formula and 0.043 sec vs 0.022 sec using the Framingham formula) (Figure 1 and Figure 2 respectively). The accuracy of the grouped measurements for all medications between observers was compared by calculating the interclass correlation coefficient (ICC). The ICC for all QT dispersion measurements showed a positive correlation of 0.34 (95% CI=0.036-0.56) between all grouped observer measurements. The ICC for QTc dispersion by the Bazett formula (0.24, 95% CI=-0.11-0.493) and the Framingham formula (0.313, 95% CI=-0.003-0.542) failed to demonstrate a significant correlation between grouped observer measurements (Table 4).

**Table 3:** QT and QTc dispersion amongst treatment groups † Denotes a significant difference between groups with p=0.006 ‡ Denotes a significant difference between groups with p=0.008 § Denotes a significant difference between groups with p=0.009 ¶Denotes a significant difference in QT dispersion between observer 2 and observer 3

	Observer 1	Observer 2	Observer 3	Average
	Mean (95% CI)	Mean (95% CI)	Mean (95% CI)	Mean (95% CI)
QT Dispersion	(seconds)			
-Amiodarone	0.057 (0.043-0.070)	0.046 (0.039-0.053)	0.046 (0.029-0.063)	0.050 (0.041-0.059)†
-Dofetilide	0.042 (0.033-0.052)	0.042 (0.035-0.048)	0.026 (0.013-0.039)	0.037 (0.031-0.043)†
-Sotalol	0.038 (0.027-0.050)	0.042 (0.036-0.047)¶	0.022 (0.008-0.035)¶	0.034 (0.027-0.040)†
QTc Dispersion (Bazett Formula)	(seconds)			
					-Amiodarone	0.061 (0.047-0.075)	0.046 (0.037-0.056)	0.050 (0.032-0.068)	0.053 (0.043-0.062)‡
-Dofetilide	0.046 (0.035-0.058)	0.044 (0.033-0.054)	0.024 (0.013-0.036)	0.038 (0.032-0.044)‡
-Sotalol	0.042 (0.030-0.055)	0.045 (0.039-0.052)¶	0.023 (0.009-0.038)¶	0.037 (0.031-0.043)‡
QTc Dispersion (Framingham Formula)	(seconds)			
-Amiodarone	0.057 (0.043-0.070)	0.045 (0.037-0.052)	0.046 (0.029-0.063)	0.049 (0.040-0.058)§
-Dofetilide	0.043 (0.033-0.053)	0.040 (0.033-0.048)	0.024 (0.012-0.036)	0.036 (0.030-0.042)§
-Sotalol	0.039 (0.028-0.051)	0.043 (0.037-0.048)¶	0.022 (0.008-0.036)¶	0.035 (0.028-0.041)§

**Table 4:** Inter-observer reliability of QTc dispersion among three different raters (n=77)

	Intraclass Correlation	95% Confidence Interval
QT Dispersion	0.034	0.036–0.056
QTc Dispersion (Bazett)	0.24	-0.11–0.493
QTc Dispersion (Framingham)	0.313	-0.003–0.542

**Figure 3:**
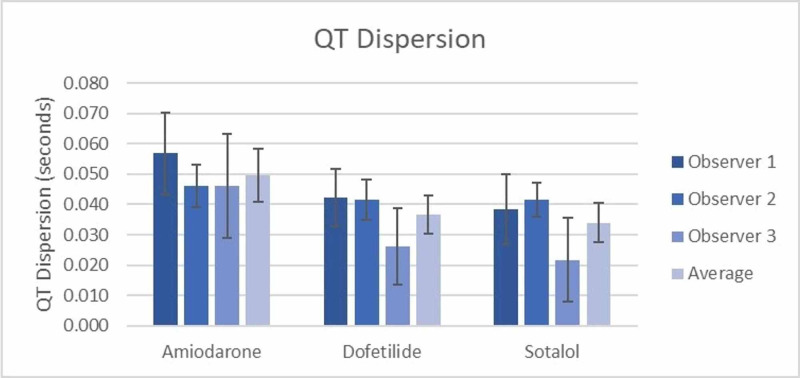
QT dispersion between groups † Denotes a significant difference between groups with p=0.006 ‡ Denotes a significant difference in QT dispersion between observer 2 and observer 3

When the measurements of QT dispersion for all observers were averaged for the amiodarone (0.050 sec), dofetilide (0.037 sec), and sotalol (0.034 sec) groups, there was a significant difference observed between groups (p=0.006) (Figure 3). Similarly, when averaging the measurements of QTc dispersion using Bazett’s, a significant difference (p=0.008) was seen between amiodarone (0.053 sec), dofetilide (0.038 sec), and sotalol (0.037 sec) (Figure 1) as well as when averaging the QTc dispersion using the Framingham formula (amiodarone 0.049 sec, dofetilide 0.036 sec, and sotalol 0.035 sec (p=0.009) (Figure 2). Notably, all three observers recorded higher QT and QTc dispersion measurements for amiodarone compared to dofetilide and sotalol (Figures 1-3).

The average measurement for QT dispersion of amiodarone between all observers was significantly higher by 0.0130 secs (p=0.038) than that of dofetilide (Figure 3), and QTc dispersion by Bazett’s similarly was 0.133 sec higher (p = 0.38) than dofetilide (Figure 1), as was the case with Framingham (0.0145 sec, p=0.026) (Figure 3). Using the average of all observers, amiodarone also had a greater QT dispersion (0.0159 sec, p=0.009) and QTc dispersion by Bazett’s (0.0145 sec, p=0.018) and Framingham (0.0156 sec, p=0.016) when compared to sotalol (Figure 1, Figure 2, and Figure 3, respectively). The differences between dofetilide and sotalol that were seen using the averages of all observers for QT and QTc dispersion using the Bazett and Framingham formulas were not significant (p = >0.05).

## Discussion

The results of this study show that the average QT/QTc dispersion measurements of all three observers were the highest in the amiodarone group as compared to dofetilide and sotalol. The QT interval measurement was also most prolonged in those patients taking amiodarone as compared to sotalol and dofetilide. The results also showed that the manual QT and QTc measurements strongly correlated with computer-derived measurements, indicating strong accuracy in the manual measurements. 

Multiple prior studies have analyzed the effect of Class III antiarrhythmics on QT dispersion, some of which, in contrast to this study's findings, have shown that amiodarone either has no effect or even reduces QT dispersion [10-12]. A notable difference between this and previous studies is the diversity of the race and ethnicity of the study population of the current study, as well as the different baseline characteristics of this study. For example, Cui et al. studied the effects of amiodarone, sematilide, and sotalol on QT dispersion and showed that amiodarone significantly reduced QT dispersion compared to sematilide and sotalol [10]. While the majority of patients in that study had coronary artery disease and a prior MI, other comorbidities and baseline electrolyte and creatinine during the study period were not reported, unlike in this study. Similarly, in Hii et al.’s study, which showed that amiodarone had no significant effect on QT dispersion, all included patients had previously been treated with Class I antiarrhythmics and had a history of polymorphic ventricular tachycardia [15].

Additionally, while studies such as Grimm et al. and Meierhenrich et al. showed no difference in QT dispersion or a predictive value in future arrhythmic events, the studies compared the same patients' ECG prior to and after initiation of amiodarone therapy [11-12]. In contrast, this study found that patients on amiodarone had a longer QT dispersion time when compared to similar patients on sotalol or dofetilide.

The medical comorbidities of patients are an important factor that may have a significant impact on QT dispersion. As shown in Table 1, patients on amiodarone had higher rates of prior MI, HFrEF, and CKD as compared to patients on dofetilide and sotalol. This difference may contribute to the study's findings, as coronary artery disease, HFrEF, and renal failure have been shown to independently increase QT and QTc dispersion [6,17-19]. The findings of this study suggest that underlying cardiac dysfunction and other comorbidities can have an additive effect on QT dispersion, which may have a synergistic effect with amiodarone, more so than other anti-arrhythmic medications.

This study, understood in the context of the above-cited literature, suggests that QT dispersion is a dynamic entity and is influenced by multiple factors. Changes in QT dispersion over various time periods have been illustrated in different populations. For example, in one study on post-myocardial infarction patients, QT dispersion time rose significantly in the first three days post-MI; however, it normalized prior to hospital discharge [20]. Similarly, the dynamic fluctuations of QT dispersion have been illustrated in patients undergoing coronary revascularization [21-22]. Medical therapy for conditions such as heart failure and hypertension has also been associated with dynamic changes in QT dispersion [23-24]. Thus, this study supports that factors aside from anti-arrhythmic medications play a role in QT dispersion.

This study also offers additional insight into the role of QT dispersion and drug-induced TdP. Class III anti-arrhythmic medications mediate their effects via interference with the myocyte action potential repolarization through the inhibition of the delayed rectifier potassium channels (Ik) [5]. This results in prolongation of action potentials, increasing myocardial cell refractoriness, and increasing the duration of the QT interval. Antiarrhythmic-associated QT prolongation increases the potential for proarrhythmic effects and fatal ventricular arrhythmias such as TdP [13]. Amiodarone has properties of all four classes of antiarrhythmics and is unique from other class III drugs because of its low frequency of drug-induced TdP [14]. The rarity of drug-induced TdP by amiodarone has been suggested to be either secondary to homogenous ventricular repolarization of all three myocardial layers, as measured by QT dispersion, or to the suppression of early after-depolarizations (EADs), which have been associated with prolonged repolarization and increased risk of ventricular arrhythmias [15-16].

Previous studies have produced limited evidence regarding the relationship between QT dispersion and drug-induced TdP, including Class III antiarrhythmics such as amiodarone, sotalol, and dofetilide [25]. Other investigations into the mechanisms for drug-induced TdP have resulted in evidence supporting EADs as a possible cause for TdP [16,26]. This study offers additional evidence that EADs rather than ventricular repolarization heterogeneity may be the mechanism for TdP. QT dispersion is a function of ventricular repolarization heterogeneity. In this study, in an ethnically diverse patient population, amiodarone had a significantly greater average measurement of QT and QTc dispersion as compared to sotalol and dofetilide. Despite the signal of an increase in ventricular repolarization heterogeneity demonstrated with amiodarone compared to the other Class II antiarrhythmics, it is well-established that the risk of Tdp is less with amiodarone as compared to other antiarrhythmics. As such, these findings suggest that the development of ventricular arrhythmias is unlikely to be due to heterogeneous ventricular repolarization alone. Rather, EADs function as either the sole nidus, or in combination with heterogeneous repolarization, to evoke the development of TdP. Previous in-vitro studies support this finding as well, as EADs induced via IKr blockade have been experimentally shown to be by sodium channel blocking Class I antiarrhythmic drugs, demonstrating a theoretic capability to prevent drug-induced TdP [27-28]. Amiodarone is unique from other class III drugs because of its additional mechanistic Class I (sodium channel inhibition) action, as well as Class II and Class IV activity, which further supports the hypothesis that amiodarone's ability to suppress EAD is responsible for the decreased incidence of TdP seen with amiodarone.

Our study had several important limitations. The retrospective nature of the study made it difficult to control for unknown confounding factors that may have influenced QT interval and QT dispersion measurements among the included patients. Additionally, there was a difference in baseline characteristics among groups, as there were a greater number of prior MI, HFrEF, and CKD patients in the amiodarone group, which could have also resulted in a greater QT and QTc dispersion in the amiodarone group. In addition, doses of various anti-arrhythmic medications were unknown at the time the included ECG was performed. As well, the study was unable to account for other medications patients were taking that may have had QT-prolonging effects. Each ECG was taken at a variable point in time when the patient was taking the anti-arrhythmic medication, and the timing of acquisition may not have been uniform among the included patients.

## Conclusions

The average QTc dispersion time was greater in patients taking amiodarone as compared to those on dofetilide and sotalol, which likely reflects a greater spatial heterogeneity of ventricular repolarization. As such, given that that amiodarone has a lower risk of TdP as compared to sotalol and dofetilide, this study suggests that QT dispersion alone is less likely to explain the increased risk of drug-induced ventricular arrhythmias such as TdP.
